# Eating habits: what foods do children between 12 and 36 months consume?

**DOI:** 10.1590/0034-7167-2022-0393

**Published:** 2023-10-09

**Authors:** Catarina Saraiva Marinho, Cândida Koch, Margarida Reis Santos

**Affiliations:** I Universidade do Porto. Porto, Portugal; II Centro Hospitalar Tondela-Viseu. Viseu, Portugal; III Escola Superior de Enfermagem do Porto. Porto, Portugal

**Keywords:** Child, Child Day Care Centers, Eating Habits, Food, Nursing, Alimentos, Enfermería, Guarderías Infantiles, Hábitos Alimentarios, Niño, Alimentos, Criança, Creches, Hábitos Alimentares, Enfermagem

## Abstract

**Objective:**

to identify toddlers’ eating habits.

**Method:**

a cross-sectional study of quantitative analysis, with a sample of 808 toddlers who attended day care centers in the district of Viseu, Portugal, between November 2018 and September 2019. Data were collected using a questionnaire directed at parents.

**Results:**

the prevalence of children who ate six meals a day was 42.8%, and 42.5%, those who ate five meals. It was found that 2.0% of children consumed chocolates, 1.0%, desserts, and 0.4%, carbonated beverages, daily. On average, dairy product (M=5.61; SD=2.62) and meat/fish/egg (M=4.80; SD=3.57) consumption was higher than recommended, while fat (M=0.48; SD=0.40), legume (M=0.49; SD=0.45), vegetable (M=1.18; SD=0.87) and water (M=0 .51; SD=0.29) consumption was lower.

**Conclusions:**

there was a higher or lower consumption than recommended for some foods, highlighting the need to implement nursing intervention programs aimed at promoting healthy eating habits in toddlers and families.

## RESUMO

**Objetivo:**

identificar os hábitos alimentares dos *toddlers*.

**Método:**

estudo transversal de análise quantitativa, com amostra de 808 *toddlers* que frequentavam creches do distrito de Viseu, Portugal, entre novembro de 2018 e setembro de 2019. Realizou-se coleta de dados com um questionário direcionado aos pais.

**Resultados:**

a prevalência de crianças que diariamente realizavam seis refeições era de 42,8%, e 42,5%, as que realizavam cinco refeições. Constatou-se que 2,0% das crianças consumia chocolates, 1,0%, sobremesas doces, e 0,4%, bebidas gaseificadas, diariamente. Em média, o consumo de laticínios (M=5,61; DP=2,62) e de carnes/peixes/ovos (M=4,80; DP=3,57) era superior ao recomendado, enquanto o consumo de gorduras (M=0,48; DP=0,40), leguminosas (M=0,49; DP=0,45), vegetais (M=1,18; DP=0,87) e de água (M=0,51; DP=0,29) era inferior.

**Conclusões:**

verificou-se um consumo superior ou inferior ao recomendado para alguns alimentos, salientando a necessidade de implementação de programas de intervenção de enfermagem que visam promover hábitos alimentares saudáveis nos *toddlers* e famílias.

## RESUMEN

**Objetivo:**

identificar los hábitos alimentarios de los niños pequeños.

**Método:**

estudio transversal con análisis cuantitativo, con una muestra de 808 niños pequeños que asistieron a guarderías en el distrito de Viseu, Portugal, entre noviembre de 2018 y septiembre de 2019. La recopilación de datos utilizó un cuestionario dirigido a los padres.

**Resultados:**

la prevalencia de niños que hacían seis comidas al día fue de 42,8%, y de 42,5%, los que hacían cinco comidas. Se encontró que el 2,0% de los niños consumía chocolates, el 1,0% postres dulces y el 0,4% bebidas carbonatadas diariamente. En promedio, el consumo de productos lácteos (M=5,61; DE=2,62) y carne/pescado/huevos (M=4,80; DE=3,57) fue superior al recomendado, mientras que el consumo de grasas (M=0,48; DE=0,40), legumbres (M=0,49; DE=0,45), hortalizas (M=1,18; DE=0,87) y agua (M=0,51; DE=0,29) fue menor.

**Conclusiones:**

hubo un consumo superior o inferior al recomendado para algunos alimentos, destacando la necesidad de implementar programas de intervención de enfermería dirigidos a promover hábitos alimentarios saludables en los toddles y las familias.

## INTRODUCTION

Toddlers - children aged between 12 and 36 months - comprise a particularly challenging age group from a food and nutritional point of view. In this phase, the changes inherent to development are directly related to nutritional intake.

According to the World Health Organization (WHO), obesity remains a current concern, being considered the “21^st^ century epidemic”“^(1)^. It is a problem to be taken into account, considering the danger it poses to people’s quality of life. In this sense, it is essential to implement political and educational actions aimed at mitigating this problem. Through health education, an attempt is made to increase information about healthy eating among the population, focusing on children and their families^(2)^. The nurses’ role stands out as a vehicle of understandable and simultaneously rigorous information, promoting adequate growth and development of a child and conscious decision-making by parents^(3)^. In health education, developed in child health consultations and focusing on parents, nurses play a fundamental role, by promoting training and enabling families to acquire skills and competencies aimed at meeting children’s needs. Many of these children spend long periods in day care. Although the management of cafeterias follows the General Directorate of Education nutritional guidelines, taking into account the constituents of meals, menus and authorized foods^(4)^, kindergarten teachers should also be considered the nurses’ focus, since they also play an important role in the development of healthy eating habits among young children.

The literature and scientific evidence have been demonstrating that childhood obesity is a socio-sanitary problem, influenced by non-modifiable risk factors (genetic factors) and by other factors on which one can and should act (unhealthy eating habits, physical inactivity, or individual and family lifestyle)^(1,5-6)^. In Portugal, the General Directorate of Health (GDH) states that “this early influence of diet on the future expression of health is called “programming”, which, as can be seen, is not only metabolic, but also behavioral”^(4)^. Inadequate feeding in toddlers can have consequences that last into adulthood. The pathogenesis of various diseases such as cardiovascular disease, obesity, diabetes mellitus, some forms of cancer, chronic respiratory diseases and musculoskeletal conditions, has been associated, among other factors, with maternal illnesses and poor infant nutrition^(6)^.

In the first 1,000 days of life, the period from conception to the end of the second year, there is great vulnerability to environmental influences. It is a unique period of opportunity to lay the groundwork for good health^(4-5)^, and food plays a decisive role in the biological evolution and development of children. Nutritional recommendations are reference values, in terms of energy and nutrients, estimated for healthy individuals. The GDH^(4)^ considers that the European Food Safety Authority (EFSA) recommendations are the most current and most appropriate for the Portuguese pediatric population.

In the first years of life, as in all other periods of the life cycle, the quality of food should predominate over the quantity of food, and it is expected that, under normal conditions, children’s appetite regulates the amount. The variety of flavors should be a constant concern throughout the child’s first years, focusing on healthy foods, restricting foods high in sugar, poor quality fats, salt and processed foods up to 12 months of age and consuming them later in moderation. The Mediterranean Diet Wheel is an excellent guide to what to offer children^(4)^.

Currently, it is known that the prevalence of overweight increases with age after five years, but, according to Rodriguez-Martinez et al.^(7)^, there are few studies that show age patterns in the prevalence of overweight in children under five years of age. It is clear, therefore, the importance of studying this age group, valuing the potential of this stage for health promotion and the acquisition of healthy lifestyle habits, presenting itself as a key factor in terms of improving the population’s future health.

## OBJECTIVE

To identify the eating habits of children between 12 and 36 months.

## METHOD

### Ethical aspects

This study is part of an investigation, within the scope of a doctoral program, and had the favorable opinion of the Ethics Committee (CETI) of *the Instituto de Ciências Biomédicas Abel Salazar, Universidade do Porto*, as well as those responsible for the day care centers involved. All parents signed an informed consent form.

### Study design, place and period

This is an exploratory study of quantitative analysis, descriptive, in a cross-sectional cohort, carried out in Private Institutions of Social Solidarity (PISS) and private day care centers in the district of Viseu, Portugal, between November 2018 and September 2019. Data collection took place in 62 PISS and 6 private day care centers.

The study was constructed according to the tool STrengthening the Reporting of OBservational studies in Epidemiology (STROBE).

### Sample, inclusion and exclusion criteria

Sample was non-probabilistic for convenience. All children, aged between 12 and 36 months, who at the time attended PISS and private day care centers in the district of Viseu, Portugal were selected. The sample was estimated considering a confidence level of 95% and a margin of error of 3% (Confidence Interval, CI), and its size (theoretical sample), of 701 participants, was considered adequate, adding 10% for flaws.

The study included children aged 12-36 months, who did not suffer from genetic or chronic diseases that could directly interfere with their nutritional status, whose parents agreed to participate in the study.

A total of 2,036 questionnaires were delivered, resulting in a final sample of 808 toddlers, corresponding to 40% of participants.

### Study protocol

The questionnaire included questions that made it possible to collect information on parents’ and children’s sociodemographic data, children’s anthropometric and clinical data, and data on the diversity and frequency of children’s eating habits. The questions about food frequency resulted from an adaptation of the “*Questionário de Frequência Alimentar e Hábitos Saudáveis dirigido a crianças dos 3 aos 7 anos*”^(8)^. Anthropometric data were collected by consulting the Child and Youth Health Bulletin for each child. The WHO anthropometric calculator (WHO Anthro v3.2.2) was used to calculate the BMI percentile of each child, based on their weight and length values.

Data referring to food frequency were obtained through open-ended questions about the amount of habitual daily consumption of foods of each of the Mediterranean Diet Wheel groups. Regarding the frequency of consumption of certain sweet and sour foods, a Likert-type scale was used.

For the instrument to meet accuracy and rigor requirements, it was important to carry out a pre-test, applying the instrument to a target group of 30 parents, with characteristics similar to the sample under study, having explained the purpose of pre-test. At the end of this stage, some questions were changed, in order to facilitate filling and understanding, such as parents’ profession, children’s age when entering the day care center, breakfast intake before going to school and the number of eggs ingested.

The intervention took place in two distinct phases. The first took place only in the municipality of Viseu, which, at the time, had a total of 40 kindergartens. Only 29 day care centers (72.5%) agreed to participate in the study. A total of 980 questionnaires were delivered, equivalent to the number of children aged between 12 and 36 months who attended day care centers. 396 were collected and 386 were validated, representing a percentage of 39.38% of the total number of participants. Due to the size of the sample obtained, a second phase followed, extending the study to the entire district of Viseu, which encompassed a total of 54 day care centers. A further 39 daycare centers agreed to participate, where 1,056 questionnaires were distributed, of which 432 were collected and 422 validated, i.e., 39.96% of questionnaires distributed in this second phase.

### Analysis of results and statistical data

Data were processed using the IBM Statistical Package for Social Sciences (SPSS), version 25. Descriptive statistics made it possible to determine absolute and percentage frequencies, some measures of central tendency, namely, averages and measures of dispersion, such as range of variation, coefficient of variation, and standard deviation, as well as measures of shape, such as skewness and flatness. As for the bivariate analysis, the chi-square test and the Fisher-Freeman-Halton test were used, applying the residual percentage test (adjusted residuals). Throughout the analysis, statistical significance was accepted for p values < 0.05 and adjusted residuals ≥1.96.

## RESULTS

The sample consisted of 808 toddlers (46.5% aged 12-23 months; 44.1% aged 24-35 months; 9.4% aged 36 months). Mothers’ ages ranged from 16 to 47 years (M=33.44; SD=5.03 years), and fathers’ ages ranged from 17 to 72 years (M=35.53; SD=5.87 years). Most fathers and mothers were between 25 and 45 years old (young adults) (91.3% vs. 94.8%, respectively). Regarding education, 53.7% of fathers had secondary education and 46.9% of mothers had higher education. In most cases, fathers and mothers were employed (92.6% vs. 85.6%, respectively). Most children (85.8%) lived in a nuclear family.

### Eating habits

The statistical analysis, regarding the number of meals eaten per day by the children, showed a minimum of four and a maximum of seven daily meals, corresponding to an average of 5.39 meals (SD=0.74 meals). There was a prevalence of children who ate six (42.8%) or five daily meals (42.5%). It should be noted that 11.1% of children ate four meals a day, a fact mainly observed in the age group between 24-35 months (13.8%). No statistically significant differences were found between the number of daily meals according to children’s age (Table 1).

**Table 1:** Number of meals eaten by children per day according to age in Viseu, Portugal, 2018-2019

Age Nº of meals	12-23 months		24-35 months		36 months		Total		Residuals			X^2^(p)
	n (376)	% (46.5)	n (356)	% (44.1)	n (76)	% (9.4)	n (808)	% (100.0)	1	2	3	
4	32	8.5	49	13.8	9	11.8	90	11.1	-2.2	2.1	0.2	10.038 (n. s.)
5	158	42.0	146	41.0	39	51.3	343	42.5	-0.2	-0.7	1.6	
6	173	46.0	149	41.9	24	31.6	346	42.8	1.7	-0.5	-2.1	
7	13	3.5	12	3.4	4	5.3	29	3.6	-0.2	-0.3	0.8	

Notes: X^2^- Chi-square test; n. s.- not significant.

The frequency of consumption of certain unhealthy foods was analyzed. As shown in Table 2, 39.2% of children did not eat chocolates, 34.5% did not eat desserts, 78.0% did not eat fast food and 88.4% did not drink carbonated beverages. However, once a week, 26.1% ate chocolate, 22.9% desserts, 1.4% fast food, and 3.3% carbonated beverages. There was also a daily consumption of chocolates by 2.0% of children, desserts by 1.0%, and carbonated beverages, by 0.4%.

**Table 2:** Frequency of consumption of certain unhealthy foods by children in Viseu, Portugal, 2018-2019

Frequency	Chocolates		Desserts		Fast food		Carbonated beverages	
	n	%	n	%	n	%	n	%
Every day	16	2.0	8	1.0	-	0.0	3	0.4
Once a week	211	26.1	185	22.9	11	1.4	27	3.3
Twice or more times a week	123	15.2	73	9.0	4	0.5	10	1.2
Once a month	84	10.4	159	19.7	126	15.6	38	4.7
Twice or more times a month	57	7.1	104	12.9	37	4.6	16	2.0
Do not eat	317	39.2	279	34.5	630	78.0	714	88.4

**Table 3:** Frequency of unhealthy food intake according to the Body Mass Index percentile of children in Viseu, Portugal, 2018-2019

BMI percentage	Weakly		Sporadically		Total		Residuals		*p* value^*^
	n	%	n	%	n	%			
Chocolates	(350)	(71.3)	(141)	(28.7)	(491)	(100.0)	1	2	
Underweight	4	1.1	-	0.0	4	0.8	1.3	-1.3	1.309 (n. s.)
Normal weight	238	68.0	99	70.2	337	68.6	-0.5	0.5	
Overweight	75	21.4	28	19.9	103	21.0	0.4	-0.4	
Obesity	33	9.4	14	9.9	47	9.6	-0.2	0.2	
Desserts	(266)	(50.3)	(263)	(49.7)	(529)	(100.0)	1	2	
Underweight	1	0.4	2	0.8	3	0.6	-0.6	0.6	1.671 (n. s.)
Normal weight	176	66.2	182	69.2	358	67.7	-0.7	0.7	
Overweight	57	21.4	55	20.9	112	21.2	0.1	-0.1	
Obesity	32	12.0	24	9.1	56	10.6	1.1	-1.1	
*Fast food*	(15)	(8.4)	(163)	(91.6)	(178)	(100.0)	1	2	
Underweight	-	0.0	2	1.2	2	1.1	-0.4	0.4	2.537 (n. s.)
Normal weight	10	66.7	108	66.3	118	66.3	0.0	0.0	
Overweight	5	33.3	35	21.5	40	22.5	1.1	-1.1	
Obesity	-	0.0	18	11.0	18	10.1	-1.4	1.4	
Carbonated beverages	(40)	(42.6)	(54)	(57.4)	(94)	(100.0)	1	2	X^2^(p)
Normal weight	23	57.5	32	59.3	55	58.5	-0.2	0.2	0.583 (n. s.)
Overweight	12	30.0	13	24.1	25	26.6	0.6	-0.6	
Obesity	5	12.5	9	16.7	14	14.9	-0.6	0.6	

Note: BMI - Body Mass Index; p-value - Fisher-Freeman-Halton Exact Test; X^2^- Chi-square test; n. s.- not significant.

Knowing that obesity is often a consequence of poor diet, we looked for the relationship between the consumption of these foods and the percentile of children’s Body Mass Index (BMI). For data analysis, the variable was recoded into: weekly consumption, which encompassed every day, once a week and two or more times a week; and sporadic consumption, which included once a month and two or more times a month, excluding those who did not consume these products. No statistically significant associations were found between the variables under study. However, it was observed that 21.0% of overweight children ate chocolates (21.4% weekly and 19.9% sporadically), as well as 9.6% of children with obesity (9.4% weekly and 9.9% sporadically). Regarding the consumption of desserts, 21.4% of overweight children and 12.0% of those with obesity consumed it weekly. Fast food was consumed by overweight (22.5%) and obese (10.1%) children. As for carbonated beverage intake, it was found that 26.6% of children who consumed it were overweight and 14.9% were obese (Table 3).

According to the appropriate daily consumption for children between 12 and 36 months of age, recommended by the GDH^(4)^ for each of the Mediterranean Diet Wheel food groups, a more significant maximum value (26 amount) was found for the dairy group (M=5.61; SD=2.62 amount), followed by the meat/fish/eggs group (24.43 amount) with an average of 4.80 (SD=3.57 amount). The lowest values were found, on average, in fat (M=0.48; SD=0.40 amount), legume (M=0.49; SD=0.45 amount) and water (M=0.51; SD=0.29 amount) consumption. It should be noted that, in all groups in the wheel, there were children who did not consume food from the same (Table 4).

**Table 4:** Food amount intake, from each group of the Mediterranean Diet Wheel, in Viseu, Portugal, 2018-2019

Variables	n	Min.	Max.	$\bar{\text{X}}$	SD	CV%		
Amount of cereals	807	0	11.43	4.07	1.62	39.85	8.62	8.26
Amount of vegetables	803	0	4.0	1.18	0.87	74.30	7.24	-1.41
Amount of fruits	805	0	6.88	2.27	0.81	35.43	5.17	23.23
Amount of legumes	807	0	2.67	0.49	0.45	92.27	1.77	17.84
Amount of dairy	805	0	26.0	5.61	2.62	46.69	18.92	46.37
Amount of meat/fish/eggs	586	0	24.43	4.80	3.57	74.36	18.88	28.38
Amount of fat	806	0	2.60	0.48	0.40	83.53	9.85	6.24
Amount of water	801	0	2.0	0.51	0.29	56.77	16.24	20.11

In order to verify the relationship between children’s age and food amount intake, the chi-square test was used. The results (Table 5) show that the majority of younger children (61.7%) eat cereal below the recommended amount as well as a large part (47.3%) of children between 24-35 months. Among older children, 47.4% eat what is recommended. There were statistically significant differences (X^2^=23.018, p=0.000), verifying the tendency of younger children to eat below recommended, of older children eating recommended and children between 24-35 months eating above recommended. Regarding vegetable consumption, there was no statistical association, but the data revealed that most children (94.4%) consumed below recommended. The same was verified in legume (84.4%), fat (91.6%) and water (89.3%) consumption. With regard to meat/fish/egg consumption, consumption above the recommended level was observed in 80.0% of children. Dairy consumption was higher than recommended in 67.1% of cases, with statistically significant differences (X^2^=43.315, p=0.000), suggesting that younger children consumed below recommended and children between 24-35 months consumed above recommended. Fruit was consumed as recommended by 78.1% of children, while 11.8% consumed less than recommended. Statistically significant differences were observed (X^2^=12.689, p=0.013), suggesting that older children ate more fruit than recommended.

**Table 5:** Amount of food, from each group of the Mediterranean Diet Wheel, eaten according to children’s age in Viseu, Portugal, 2018-2019

Age Variables	12-23 months		24-35 months		36 months		Total		Residuals			X^2^(p)
	n	%	n	%	n	%	n	%				
Amount of cereals	(376)	(46.5)	(355)	(44.0)	(76)	(9.4)	(807)	(100.0)	1	2	3	
Below recommended	232	61.7	168	47.3	30	39.5	430	53.3	4.5	-3.0	-2.5	23.018 (^***^)
Recommended	112	29.8	136	38.3	36	47.4	284	35.2	-3.0	1.6	2.3	
Above recommended	32	8.5	51	14.4	10	13.2	93	11.5	-2.5	2.2	0.5	
Amount of vegetables	(373)	(46.5)	(355)	(44.2)	(75)	(9.3)	(803)	(100.0)	1	2	3	
Below recommended	351	94.1	336	94.6	71	94.7	758	94.4	-0.3	0.3	0.1	0.114 (n. s.)
Recommended	22	5.9	19	5.4	4	5.3	75	5.6	0.3	-0.3	-0.1	
Amount of dairy	(373)	(46.3)	(356)	(44.2)	(76)	(9.4)	(805)	(100.0)	1	2	3	
Below recommended	54	14.5	9	2.5	-	0.0	63	7.8	6.5	-5.0	-2.7	43.315 (^***^)
Recommended	89	23.9	93	26.1	20	26.3	202	25.1	-0.7	0.6	0.3	
Above recommended	230	61.7	254	71.3	56	73.7	540	67.1	-3.0	2.3	1.3	
Amount of fruits	(374)	(46.5)	(355)	(44.1)	(76)	(9.4)	(805)	(100.0)	1	2	3	
Below recommended	52	19.3	36	10.1	7	9.2	95	11.8	1.7	-1.3	-0.7	12.689 (^*^)
Recommended	296	79.1	278	78.3	55	72.4	629	78.1	0.6	0.1	-1.3	
Above recommended	26	7.0	41	11.5	14	18.4	81	10.1	-2.7	1.2	2.5	
Amount of legumes	(375)	(46.5)	(356)	(44.1)	(76)	(9.4)	(807)	(100.0)	1	2	3	
Below recommended	325	86.7	295	82.9	61	80.3	681	84.4	1.7	-1.1	-1.0	5.543 (n. s.)
Recommended	27	7.2	33	9.3	11	14.5	71	8.8	-1.5	0.4	1.8	
Above recommended	23	6.1	28	7.9	4	5.3	55	6.8	-0.7	1.1	-0.6	
Amount of meat/fish/eggs	(267)	(45.7)	(262)	(44.9)	(55)	(9.4)	(584)	(100.0)	1	2	3	
Below recommended	21	7.9	22	8.4	4	7.3	47	8.0	-0.1	0.3	-0.2	6.311 (n. s.)
Recommended	41	15.4	26	9.9	3	5.5	70	12.0	2.3	-1.4	-1.6	
Above recommended	205	76.8	214	81.7	48	87.3	467	80.0	1.8	0.9	1.4	
Amount of fat	(376)	(46.7)	(355)	(44.0)	(75)	(9.3)	(806)	(100.0)	1	2	3	*p* value^*^
Below recommended	353	93.9	319	89.9	66	88.0	738	91.6	2.2	-1.5	-1.2	7.869 (n. s.)
Recommended	5	1.3	3	0.8	1	1.3	9	1.1	0.5	-0.7	0.2	
Above recommended	18	4.8	33	9.3	8	10.7	59	7.3	-2.6	1.9	1.9	
Amount of water	(373)	(46.3)	(356)	(44.2)	(76)	(9.4)	(805)	(100.0)	1	2	3	X^2^(p)
Below recommended	341	91.7	312	88.4	62	81.6	715	89.3	2.0	-0.7	-2.3	7.345 (n. s.)
Recommended	21	5.6	26	7.4	9	11.8	56	7.0	-1.4	0.4	1.7	
Above recommended	10	2.7	15	4.2	5	6.6	30	3.7	-1.5	0.7	1.4	

Note: p-value^*^- Fisher-Freeman-Halton Exact Test; X2- Chi-square test;

*** p<0.001;

* p<0.05; n. s.- not significant.

## DISCUSSION

As the main conclusions of this study, we can say that there was an adequate number of meals per day, but an inadequate consumption of certain unhealthy foods, such as chocolates, carbonated beverages or fast food by children, which includes, although without statistical significance, overweight and obese children. Consumption above or below the recommended food intake of some of the Mediterranean Diet Wheel groups was also determined.

According to scientific evidence, the first three years of life are a sensitive period for the development of perception, cognition, behaviors and experiences in relation to food^(9)^. Some studies^(10-11)^ revealed fast food, high energy density food (such as desserts, sweets, chocolates) and carbonated beverage consumption in a significant sample of children, particularly between two and three years old.

The data from the present study corroborate those found by other investigators^(12)^, in a systematic literature review on dietary patterns in children between two and five years of age, which found a consumption of potentially obesogenic foods, increasing the risk of children becoming overweight. The consumption of these foods is not prohibited, but must be limited in quantity and frequency^(4)^. Increasingly, the media and the accelerated pace of everyday life can boost the consumption of some of these foods, with family education being of paramount importance, to allow the child and family to understand the advantages of correct eating behavior.

Free sugar or sugar-sweetened beverage intake as a determinant of body weight, a parameter assessed in the present study, has become the focus of relevant research. As preferred options for snacks, sugary drinks and foods are the greatest source of added sugars and contribute a significant amount of calories to children’s diets^(13)^. There are several researchers who approach this topic in their investigations. In a cross-sectional study, with a sample of 4,839 children aged less than two years, the authors found that sugary drink intake was present in 32.0% of children, revealing a high prevalence of sweet drink consumption by children aged up to two years^(14)^, a fact also verified in our study.

According to our data, consumption responsible for this intake included sugary drinks, sugary breakfast cereals, cookies, sweets, chocolate and sugar. Conclusions similar to those of other researchers^(15-16)^, reporting that consuming fruit juice, breakfast cereals, desserts and soft drinks begins after the first year of life, reveal that at 30 months of age 50.0% of children had already tasted some type of sugary drink^(15)^ and that 99.0% of children aged 6 to 24 months consumed some sugar daily^(16)^. In a study carried out with 1,824 American children, from birth to 23 months of age, the authors concluded that 58.9% of children aged between 12 and 18 months and 62.9% of those aged between 19 and 23 months habitually consumed flavored milk and sugar-sweetened beverages^(17)^. Other investigators^(18)^ found that the median usual intake of free sugars at two years of age was 22.5 g/day, contributing with a median of 8.0% in the estimated energy requirement.

The Portuguese National Program for Healthy Eating Promotion (PNPA - *Programa Nacional para a Promoção da Alimentação Saudável*) of the GDH^(19)^ indicates that sugary drinks have increasingly gained prominence in the dietary pattern of Portuguese children, verifying that 22.0% of children consume daily soft drinks or juices (≥220 g/day). As shown in the aforementioned studies, the main sources of free sugars were non-essential foods, such as sparkling fruit juices, biscuits, cakes, desserts and sweets. With regard to free sugars, the EFSA does not set an upper limit, however it warns that high sugar consumption increases the risk of cavities^(20)^.

Regarding the various food groups, according to the Mediterranean Diet Wheel, the children in our sample revealed the maximum and most significant value (26 amount) in the consumption of dairy products and meat/fish/eggs (24.43 amount). The lowest values were found, on average, in fat, legume and water consumption. According to GDH^(6)^, children in this age group should consume 3-4 amounts of dairy products daily; 1.5-2 amount of meat, fish or eggs; 4-6 amount of cereals, derivatives and tubers; 3-4 amount of vegetables; 2-3 amount of fruit; 1 portion of oils and fats; and 1 liter of water, while legumes should be consumed 3 times a week. According to these recommendations, in this study, only fruit consumption was found to be adequate.

A study^(21)^ based on data from the Feeding Infants and Toddlers Study 2008 (FITS 2008), in a random sample of 1,323 children aged between two and three years, found a prevalence of habitual food intake that was inconsistent with the recommendations, the highest being for vegetables (91.0%), whole grains (94.0%) and fats (>99.0%), eating protein and dairy products below recommended (32.0% and 48.0%, respectively).

In the children participating in this study, the consumption of dairy products was above the recommended level, and that of vegetables, below the recommended level. This corroborates the conclusions of another study, carried out in Portugal, on the eating patterns of toddlers^(22)^, which found a consumption of fruit and vegetables lower than recommended and a consumption of dairy products considerably higher. These results may suggest a possible overestimation of dairy product consumption and a non-appreciation of vegetable consumption by parents of toddlers. Low fruit consumption is a problem found in children in several countries^(23-24)^. However, the results of our study do not prove this evidence, as most children consumed an adequate amount of fruit.

The food offer for toddlers should be based on variety, justifying the choice of foods that are part of the food chain and the Diet Wheel or the Mediterranean Diet Wheel^(25)^. The results found in this study reinforce that a varied offer should be favored in relation to the various food groups and the constituents of each one of them, always bearing in mind that quality/variety matters rather than quantity. It is ideal that the repeated offer of each new food occurs in the family and school, which will facilitate its acceptance by the child, in line with the Portuguese Ministry of Health guidelines^(4)^.

### Study limitations

As possible limitations of this study, we highlight the fact that information was only collected on cooked vegetable consumption, not specifying the consumption of soup, which is very common in this age group. Data collection carried out through a self-report questionnaire was a limitation, thus depending on parents’ honesty, and may be influenced by behaviors that they recognize as socially preferable. Another limitation is related to the fact that the study, not being longitudinal, does not allow assessing the future consequences of less adequate eating habits in the studied children.

### Contributions to nursing

This study highlights the need to carry out more research on toddlers’ eating behaviors, in order to obtain information that will help design nursing interventions and policies to prevent diseases related to toddlers’ diets. Nurses play a key role in these interventions, as health education actions are one of the basic interventions in child health consultations, contributing to the empowerment of parents by raising awareness and improving their knowledge.

## CONCLUSIONS

The results found in the present study allow us to say that, although the majority of the sample (85.3%) complied with the GDH recommendations with regard to the number of meals to be had daily (42.5% five meals; 42.8% six meals), its constitution may not be the most adequate. There was an insufficient or excessive consumption of some foods from the various constituent groups of the Mediterranean Diet Wheel, in addition to the consumption of sweets and fast food not recommended for children in this age group.

In the promotion and greater focus on education for toddlers’ nutritional health, emphasis is placed on nurses’ intervention, following the GDH general and guiding principles with regard to meeting the nutritional needs of this age group. It is a phase of excellence for “learning to eat” and establishing good eating habits, a practice seen as fundamental and urgent. Thus, it is essential that interventions include caregivers (family and school) so that there is co-responsibility for dietary rigor and healthier eating practices. Families exert a great influence on children’s overall health, as they are their first learning environment, functioning as models. At the dietary level, parents are the first examples, with a direct influence on children’s eating behavior, making it important for them to be aware of the evolution of these behaviors during the first years of life as biological and behavioral processes aimed at meeting children’s health and growth requirements.
